# A novel flexible exponent power-X family of distributions with applications to COVID-19 mortality rate in Mexico and Canada

**DOI:** 10.1038/s41598-024-59720-1

**Published:** 2024-04-18

**Authors:** Zubir Shah, Dost Muhammad Khan, Imad Khan, Bakhtiyar Ahmad, Mouna Jeridi, Sanaa Al-Marzouki

**Affiliations:** 1 Abdul Wali Khan University Mardan, Mardan, Pakistan; 2 Higher Education Department Afghanistan, Kart-e-Char, Afghanistan; 3 Biology Department, College of Science, King Khalid University, 61413 Abha, Saudi Arabia; 4 Statistics Department, Faculty of Science, King Abdul Aziz University Jeddah, Jeddah, Kingdom of Saudi Arabia

**Keywords:** Novel Generalized Exponent Power X family, Monte Carlo simulation, Weibull distribution, COVID-19 data, Maximum likelihood estimation, Diseases, Health care, Engineering, Mathematics and computing

## Abstract

This paper aims to introduce a novel family of probability distributions by the well-known method of the T–X family of distributions. The proposed family is called a “Novel Generalized Exponent Power X Family” of distributions. A three-parameters special sub-model of the proposed method is derived and named a “Novel Generalized Exponent Power Weibull” distribution (NGEP-Wei for short). For the proposed family, some statistical properties are derived including the hazard rate function, moments, moment generating function, order statistics, residual life, and reverse residual life. The well-known method of estimation, the maximum likelihood estimation method is used for estimating the model parameters. Besides, a comprehensive Monte Carlo simulation study is conducted to assess the efficacy of this estimation method. Finally, the model selection criterion such as Akaike information criterion (AINC), the correct information criterion (CINC), the Bayesian information criterion (BINC), the Hannan–Quinn information criterion (HQINC), the Cramer–von-Misses (CRMI), and the ANDA (Anderson–Darling) are used for comparison purpose. The comparison of the NGEP-Wei with other rival distributions is made by Two COVID-19 data sets. In terms of performance, we show that the proposed method outperforms the other competing methods included in this study.

## Introduction

In the literature on distribution theory, the researchers have proposed many probability distributions for analyzing and predicting real-world phenomena but the real-world phenomena are complex and complicated. Therefore, no particular probability distribution is yet proposed to handle (for analyzing and predicting) every phenomenon. Similarly, in the literature of distribution theory, the exponential and Rayleigh distributions are the most popular and well-known distributions and are widely used in lifetime analysis. However, when the real-life phenomena are complex then these probability distributions are not suitable for accurate representation of the data. For example, the exponential distribution is concerned with describing data with only a constant failure (or hazard) rate function. On the other hand, the Rayleigh distribution is used to model data that have only an increasing failure rate function. Furthermore, the Weibull distribution is also considered one of the most important lifetime distributions, which has both the capability of the exponential and Rayleigh distributions and offers data modeling that has increasing, decreasing, and constant failure rate functions. However, in many applied fields, especially, in biomedical and engineering areas the behaviour of hazard function changes with time non-monotonically. So, in such phenomena, the Weibull distribution is not a suitable choice to implement; see (Almalki and Yuan^1^) for more reading. To deal with such difficulties, generalized versions of these classical models are needed. To this end, the researchers are trying to derive new methods (new family of distributions) to obtain the generalized version of the classical distributions with greater distributional flexibility. Most of the new methods in the literature are developed by adding one or more additional parameters to the baseline distributions (or existing distributions) to obtain new updated versions of these existing distributions that are analytically more flexible in modeling as a practical viewpoint; see (Usman et al.^2^) for more reading. In the recent past, several families of probability distributions have been proposed in the literature of distributions theory, for example, Mudholkar and Srivastava^3^ proposed a very simple approach called the Exponentiated family of distributions. The proposed method is based on inserting only one additional parameter into the baseline distributions. The CDF (cumulative distribution function) of the exponentiated family is given by

1 $$Y\left( {x;\phi ,\mu } \right) = \left[ {A\left( {x;\mu } \right)} \right]^{\phi } ,\quad \phi ,\mu > 0,\quad x \in {\mathbb{R}},$$

where, $\phi > 0$ is an extra (or additional) shape parameter and $A\left( {x;\mu } \right)$ is the CDF of any baseline random variable depending on parameter vector $\mu$. Marshal and Olkin^4^ introduced a new method for obtaining the modified version of the existing distributions. Their suggested method is called, the Marshal and Olkin family of distributions. The CDF of the Marshal Olkin family is defined by

2 $$Y\left( {x;\phi ,\mu } \right) = \frac{{A\left( {x;\mu } \right)}}{{1 - (1 - \phi )\left[ {1 - A\left( {x;\mu } \right)} \right]}},\;\;\phi ,\mu > 0,\;x \in {\mathbb{R}},$$

Using Eq. (2), Marshal and Olkin^4^ derived two special sub-models namely, Marshal–Olkin exponential and Marshal–Olkin Weibull distributions. Latterly, the authors used Eq. (2), and several probability distributions have been proposed in the literature, see (Ghitany et al.^5^, Gui et al.^6^, and Saboor et al.^7^) for more reading.

Similarly, in this regard, Mahdavi and Kundu^8^ also proposed a new family of distributions by incorporating one additional parameter to the baseline distribution. They named their proposed method by Alpha Power transformation (APTra) family of distributions. The CDF of the APTra family is defined by

3 $$Y\left( {x;\alpha_{1} ,\mu } \right) = \frac{{\alpha_{1}^{{A\left( {x;\mu } \right)}} - 1}}{{\alpha_{1} - 1}},\;\alpha_{1} ,\;\;\mu > 0,\;\;\alpha_{1} \ne 1,\;\;x \in {\mathbb{R}},$$

Using Eq. (3), Mahdavi and Kundu^8^ modified the exponential distribution and named the alpha power transformed exponential (APTra-Expo) distribution. Furthermore, considering Eq. (3), various contributions have been made in the literature on distribution theory; see Dey et al.^9^, Ihtisham et al.^10^, and Hassan et al.^11^.

In the recent past, Shah et al.^12^ proposed a new method of probability distribution by incorporating one additional parameter into baseline distribution. Their proposed method is called, the new generalized logarithmic–X (NGLog–X) family of distributions. The CDF of the NGLog-X family is given by

4 $$Y\left( {x;\phi ,\mu } \right) = \frac{{e^{\phi } A\left( {x;\mu } \right)}}{{\left[ {e - \log A\left( {x;\mu } \right)} \right]^{\phi } }},\;\;\phi ,\mu > 0,\;\;x \in {\mathbb{R}},$$

Using Eq. (4), Shah et al.^12^ modified the Weibull distribution and named a new generalized logarithmic Weibull (NGLog-Wei) distribution. For recent developments about the distributional approaches, we refer to a superior extension for the Lomax distribution with application to Covid-19, proposed by Alsuhabi et al.^13^, a novel logarithmic approach to generate new probability distributions, proposed by Zhao et al.^14^, a novel updated-W family of distributions, proposed by Alnssyan et al.^15^, a new Type 1 Alpha Power family of distributions, proposed by Tekle et al.^16^, the Type II-Topp-Leone-Gompertz-G family of distributions with applications to COVID-19 data, proposed by Chamunorwa et al.^17^, a Weighted Cosine-G family of distributions, proposed by Odhah et al.^18^, some inferences on three parameters Birnbaum–Saunders distribution, developed by Shakil et al.^19^, a statistical analysis of excess mortality mean at Covid-19 in 2020–202, proposed by Raihen et al.^20^, exponentiated generalized Weibull exponential distribution, proposed by Abonongo et al.^21^, a case study for Kuwait mortality during the consequent waves of COVID-19, derived by BuHamra et al.^22^, and a new inverse Rayleigh distribution with applications of COVID-19 data, developed by El-Sherpieny et al.^23^.

In this research paper, taking motivation from the above discussion, we also propose a new method for obtaining more flexible probability distributions. The proposed method is obtained by implementing the T–X family approach proposed by (Alzaatreh et al.^24^). The proposed method may be named a Novel Generalized Exponent Power-X (NGEP-X) family of distributions. Based on the NGEP-X method, the improvised version of the Weibull distribution with distributional flexibility in shapes of PDF (probability density function) and HF (hazard function) is introduced. Based on COVID-19 data sets, the fitting power of the proposed work is compared with Alpha Power Transformed Weibull, New Reduce Logarithmic Weibull, Kumaraswamy Weibull, Weibull, Marshal Olkin Nadarajah Haghigh, and Gull Alpha Power Weibull distributions; see Table 3, for references of these competing distributions.

The rest of the work done in the study is organized into seven sections: In section "NGEP-X family", the newly proposed family of distributions is comprehensively derived. Section "NGEP-Wei distribution", gives a special sub-model of the proposed family, named a Novel Exponent power Weibull distribution, and in the same section, the shapes of its CDF, survival function, PDF, and HF are also graphically illustrated. The mathematical properties of the NGEP-X family are given (or derived) in Section "Basic mathematical properties". The method of Maximum Likelihood Estimation is applied for estimating the model parameters of the proposed method in section "Estimation, experiment and simulation". Practical applications via two COVID-19 data sets (describing the mortality rates of the countries of Canada and Mexico) are discussed in section 6. Finally, section 7 gives the "Concluding remarks" based on the analyses done in this paper.

## NGEP-X family

In this section, the CDF, PDF, SF (survival function), HF, and CHF (cumulative hazard function) of the NGEP-X family of distributions are computed.

### Definition

Let $m\left( t \right) = e^{ - \alpha t}$ be the PDF of exponential random variable, say *T*, where $T \in \left[ {b_{1} ,b_{2} } \right]$ for $- \infty \le b_{1} < b_{2} \le \infty$ and let suppose $K\left[ {A\left( {x;\mu } \right)} \right]$ be a function of CDF $A\left( {x;\mu } \right)$ of a random variable, say X, satisfying the following three conditions.

I. $$K\left[ {A\left( {x;\mu } \right)} \right] \in \left[ {b_{1} ,b_{2} } \right]$$,
II. $K\left[ {A\left( {x;\mu } \right)} \right]$ is monotonically increasing function and differentiable,
III. $K\left[ {A\left( {x;\mu } \right)} \right] \to b_{1}$ as $x \to - \infty$ and $K\left[ {A\left( {x;\mu } \right)} \right] \to b_{2}$ as $x \to \infty$.

According to (Alzaatreh et al.^24^) the CDF of T–X family defined by

5 $$Y\left( {x;\mu } \right) = \int_{{b_{1} }}^{{K[A\left( {x;\mu } \right)\,]}} {m\left( t \right)dt} = M\left( {K\left[ {A(x;\mu )} \right]} \right),\;\;x \in {\mathbb{R}},$$

where, $K\left[ {A(x;\mu )} \right]$ satisfies the conditions (I)–(III); see (Alzaatreh et al.^24^). The PDF $Y\left( {x;\mu } \right)$ of T–X distribution, associated with Eq. (5) is as follow

6 $$y\left( {x;\mu } \right) = m\left( {K\left[ {A\left( {x;\mu } \right)} \right]} \right)\frac{d}{dx}\left\{ {K\left[ {A\left( {x;\mu } \right)} \right]} \right\},\;\;x \in {\mathbb{R}}.$$

Now, by using $m\left( t \right) = e^{ - t}$ as the PDF of exponential distribution with rate parameter $\left( {\alpha = 1} \right)$ and setting the upper limit $K\left[ {A(x;\mu )} \right] = - \log \left( {e^{{\phi A\left( {x;\mu } \right)^{2} }} - e^{\phi } A(x;\mu )^{2} } \right)$ and lower limit $b_{1} = 0$ in Eq. (5), we get the CDF $Y\left( {x;\phi ,\mu } \right)$ of the NGEP-X family, which is given by

7 $$Y\left( {x;\phi ,\mu } \right) = 1 - \left( {e^{{\phi A\left( {x;\mu } \right)^{2} }} - e^{\phi } A(x;\mu )^{2} } \right),\;\;\phi \in {\mathbb{R}}^{ + } ,\;\;x \in {\mathbb{R}}.$$

where, $A\left( {x;\mu } \right)$ is the CDF of any sub-model which may be depending on $\mu \in {\mathbb{R}}$. To prove that whether the CDF $Y\left( {x;\phi ,\mu } \right)$ is an exact CDF or not, we have the following two proposition.

### Proposition 1

The CDF $Y\left( {x;\phi ,\mu } \right)$ derived in Eq. (7), we need to prove.

$$\mathop {\lim }\limits_{x \to - \infty } Y\left( {x;\phi ,\mu } \right) = 0,\;{\text{and}}\;\mathop {\lim }\limits_{x \to \infty } Y\left( {x;\phi ,\mu } \right) = 1.$$

### Proof

$$\begin{aligned} & \mathop {\lim }\limits_{x \to - \infty } Y\left( {x;\phi ,\mu } \right) = \mathop {\lim }\limits_{x \to - \infty } \left\{ {1 - \left( {e^{{\phi A\left( {x;\mu } \right)^{2} }} - e^{\phi } A(x;\mu )^{2} } \right)} \right\}, \\ & \quad = 1 - \left( {e^{{\phi A\left( { - \infty ;\mu } \right)^{2} }} - e^{\phi } A( - \infty ;\mu )^{2} } \right) = 0. \\ \end{aligned}$$

and

$$\begin{aligned} & \mathop {\lim }\limits_{x \to \infty } Y\left( {x;\phi ,\mu } \right) = \mathop {\lim }\limits_{x \to \infty } \left\{ {1 - \left( {e^{{\phi A\left( {x;\mu } \right)^{2} }} - e^{\phi } A(x;\mu )^{2} } \right)} \right\}, \\ & \quad = 1 - \left( {e^{{\phi A\left( {\infty ;\mu } \right)^{2} }} - e^{\phi } A(\infty ;\mu )^{2} } \right) = 1 \\ \end{aligned}$$

### Proposition 2

The CDF $Y\left( {x;\phi ,\mu } \right)$ derived in Eq. (7), is RC (right continues) and differentiable.

$$\frac{d}{dx}Y\left( {x;\phi ,\mu } \right) = y\left( {x;\phi ,\mu } \right).$$

Hence, from proposition 1 and 2, we concluded that the CDF $Y\left( {x;\phi ,\mu } \right)$ in Eq. (7) is a valid CDF. Corresponding to Eq. (7), the PDF $y\left( {x;\phi ,\mu } \right)$ of the NGEP-X family is given by

8 $$y\left( {x;\phi ,\mu } \right) = 2a\left( {x;\mu } \right)A\left( {x;\mu } \right)\left[ {e^{\phi } - \phi e^{{\phi A\left( {x;\mu } \right)\,}} } \right],\;\;\mu \in {\mathbb{R}},\;\;x \in {\mathbb{R}}.$$

where,$\frac{d}{dx}A\left( {x;\mu } \right) = a\left( {x;\mu } \right)$ and the rest of the SF $S\left( {x;\phi ,\mu } \right)$, HF $h\left( {x;\phi ,\mu } \right)$, and CHF $H\left( {x;\phi ,\mu } \right)$ of the NGEP-X family are respectively given by

9 $$S\left( {x;\phi ,\mu } \right) = \left( {e^{{\phi A\left( {x;\mu } \right)^{2} }} - e^{\phi } A(x;\mu )^{2} } \right),\;\;\mu \in {\mathbb{R}},\;\;x \in {\mathbb{R}},$$

10 $$h\left( {x;\phi ,\mu } \right) = 2a\left( {x;\mu } \right)A\left( {x;\mu } \right)\frac{{\left( {e^{\phi } - \phi e^{{\phi A\left( {x;\mu } \right)^{2} }} } \right)}}{{\left( {e^{{\phi A\left( {x;\mu } \right)^{2} }} - e^{\phi } A\left( {x;\mu } \right)^{2} } \right)}},\;\;\mu \in {\mathbb{R}},\;\;x \in {\mathbb{R}},$$

and

11 $$H\left( {x;\phi ,\mu } \right) = - \log \left( {e^{{\phi A\left( {x;\mu } \right)^{2} }} - e^{\phi } A(x;\mu )^{2} } \right),\;\;\mu \in {\mathbb{R}},\;\;x \in {\mathbb{R}},$$

## NGEP-Wei distribution

This section of the article is based on a three-parameter-specific sub-model of the NGEP-X family of distributions. This special model of the proposed family is called a Novel Generalized Exponent Power Weibull distribution (NGEP-Wei for short). Let $A(x;\mu )$ and $a(x;\mu )$ be the corresponding CDF and PDF of the classical Weibull distribution expressed as $A(x;\mu ) = 1 - e^{{ - \alpha x^{\delta } }}$ and $a(x;\mu ) = \alpha \delta x^{\delta - 1} e^{{ - \alpha x^{\delta } }}$, respectively, ($\alpha ,\delta ,x \in {\mathbb{R}}^{ + }$), where $\mu = \left( {\alpha ,\delta } \right)$. Using $A(x;\mu ) = 1 - e^{{ - \alpha x^{\delta } }}$ in Eq. (7), we obtain the updated version of the Weibull distribution. The CDF $Y(x;\phi ,\mu )$, and SF $S(x;\phi ,\mu )$ of the NGEP-Wei distribution (or updated version) is given by the following form, respectively

12 $$Y\left( {x;\phi ,\mu } \right) = 1 - \left( {e^{{\phi \left( {1 - e^{{ - \alpha x^{\delta } }} } \right)^{2} }} - e^{\phi } \left( {1 - e^{{ - \alpha x^{\delta } }} } \right)^{2} } \right),\;\;\phi ,\alpha ,\delta \in {\mathbb{R}}^{ + } ,\;\;x \in {\mathbb{R}}^{ + } ,$$

and

13 $$S\left( {x;\phi ,\mu } \right) = \left( {e^{{\phi \left( {1 - e^{{ - \alpha x^{\delta } }} } \right)^{2} }} - e^{\phi } \left( {1 - e^{{ - \alpha x^{\delta } }} } \right)^{2} } \right),\;\;\phi ,\alpha ,\delta \in {\mathbb{R}}^{ + } ,\;\;x \in {\mathbb{R}}^{ + } .$$

**Figure 1:**
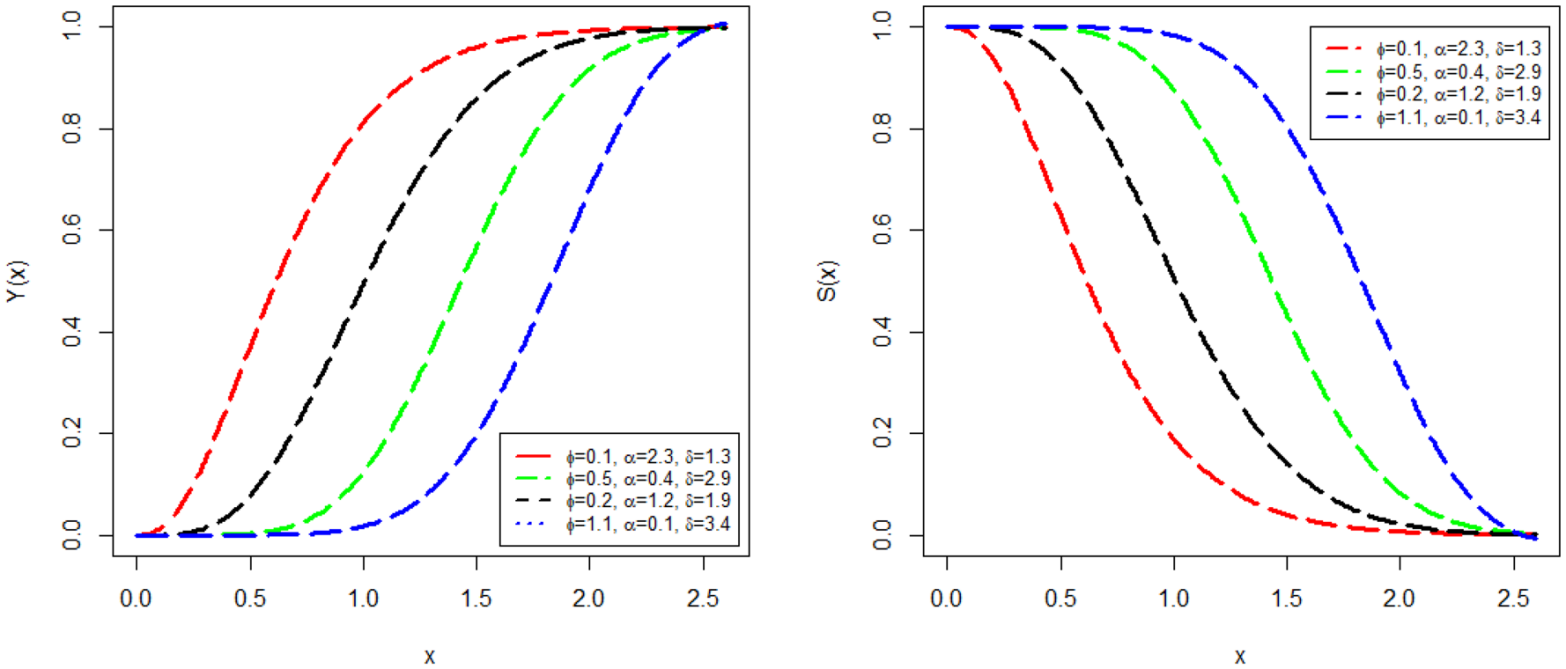
$Y(x;\phi ,\mu )$ and $S(x;\phi ,\mu )$ graphs with different parameters values.

Some attractive plots of $Y(x;\phi ,\mu )$ and $S(x;\phi ,\mu )$ are visualized in Fig. 1. The plots of CDF $Y(x;\phi ,\mu )$ and SF $S(x;\phi ,\mu )$ are obtained with different parameters values (i) $\phi =$ 0.1, $\alpha =$ 2.3, and $\delta =$ 1.3 (red line), (ii) $\phi =$ 0.5, $\alpha =$ 0.4, and $\delta =$ 2.9 (green line), (iii) $\phi =$ 0.2, $\alpha =$ 1.2, and $\delta =$ 1.9 (black line), and (iv) $\phi =$ 1.1, $\alpha =$ 0.1, and $\delta =$ 3.4 (blue line). From Fig. 1, it is visually confirmed that the proposed model has a valid CDF.

Furthermore, the PDF $y(x;\phi ,\mu )$ , HF $h(x;\phi ,\mu )$ and function for CHF $H(x;\phi ,\mu )$ corresponding to Eq. (12) are given in Eqs. (14)–(16), respectively, by

14 $$y(x;\phi ,\mu ) = 2\alpha \delta x^{\delta - 1} e^{{ - \alpha x^{\delta } }} \left( {1 - e^{{ - \alpha x^{\delta } }} } \right)\left( {e^{\phi } - \phi e^{{\phi \left( {1 - e^{{ - \alpha x^{\delta } }} } \right)^{2} }} } \right),\;\;x \in {\mathbb{R}}^{ + } ,$$

15 $$h(x;\phi ,\mu ) = 2\alpha \delta x^{\delta - 1} e^{{ - \alpha x^{\delta } }} \left( {1 - e^{{ - \alpha x^{\delta } }} } \right)\frac{{\left( {e^{\phi } - \phi e^{{\alpha \left( {1 - e^{{ - \alpha x^{\delta } }} } \right)^{2} }} } \right)}}{{\left( {e^{{\phi \left( {1 - e^{{ - \alpha x^{\delta } }} } \right)^{2} }} - e^{\phi } \left( {1 - e^{{ - \alpha x^{\delta } }} } \right)^{2} } \right)}},\;\;x \in {\mathbb{R}}^{ + } ,$$

and

16 $$H(x;\phi ,\mu ) = - \log \left( {e^{{\phi \left( {1 - e^{{ - \alpha x^{\delta } }} } \right)^{2} }} - e^{\phi } \left( {1 - e^{{ - \alpha x^{\delta } }} } \right)^{2} } \right),\;\;x \in {\mathbb{R}}^{ + } .$$

**Figure 2:**
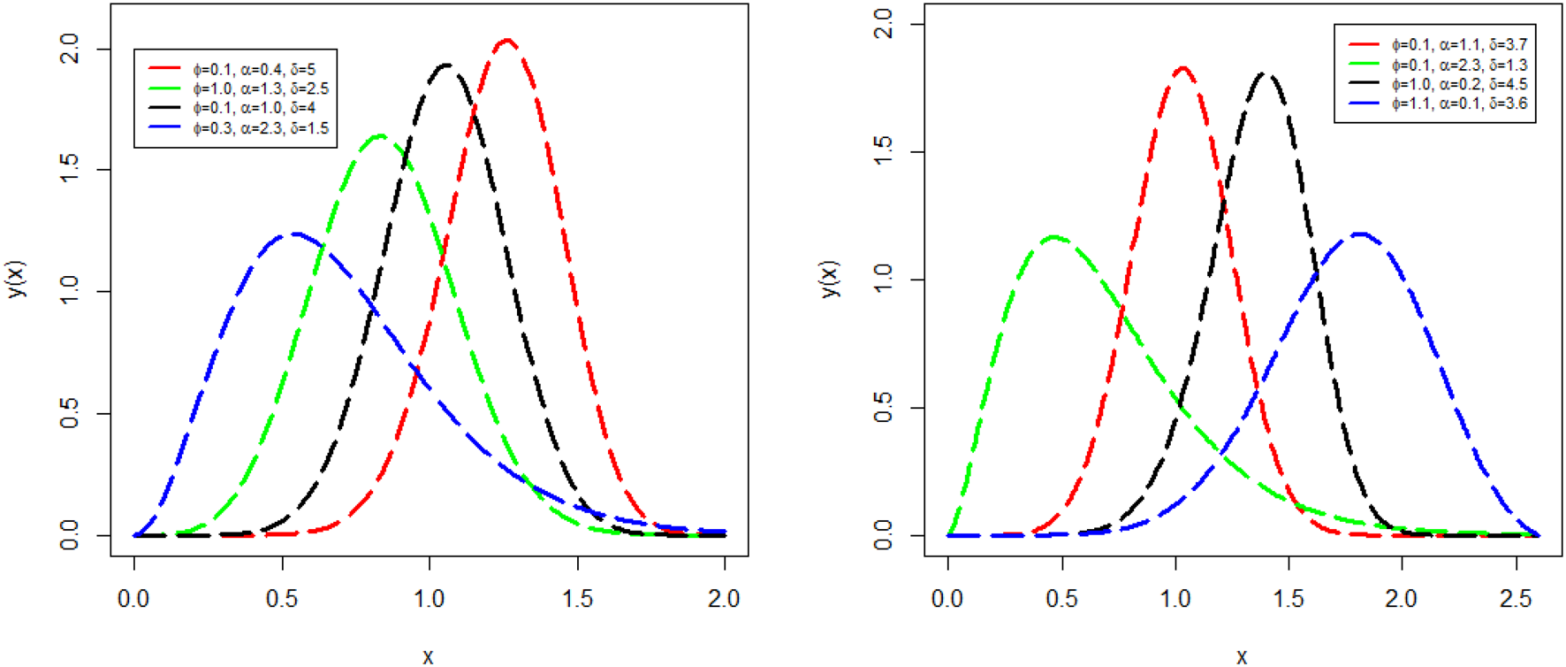
Some different plots of $y(x;\phi ,\mu )$ with different parameters values of NGEP-Wei distribution.

Some attractive, right skewed, lift skewed, and symmetrical PDF $y(x;\phi ,\mu )$ plots are visualized in Fig. 2. The plots in lift penal of the Fig. 2 of $y(x;\phi ,\mu )$ are obtained with different parameters values (i) $\phi =$ 0.1, $\alpha =$ 0.4, and $\delta =$ 5.0 (red line), (ii) $\phi =$ 1.0, $\alpha =$ 1.3, and $\delta =$ 2.5 (green line), (iii) $\phi =$ 0.1, $\alpha =$ 1.0, and $\delta =$ 4.0 (black line), and (iv) $\phi =$ 0.3, $\alpha =$ 2.3, and $\delta =$ 1.5 (blue line). The plots in right penal of the Fig. 2 of $y(x;\phi ,\mu )$ are obtained with different parameters values (i) $\phi =$ 0.1, $\alpha =$ 1.1, and $\delta =$ 3.7 (red line), (ii) $\phi =$ 0.1, $\alpha =$ 2.3, and $\delta =$ 1.3 (green line), (iii) $\phi =$ 1.0, $\alpha =$ 0.2, and $\delta =$ 4.5 (black line), and (iv) $\phi =$ 1.1, $\alpha =$ 0.1, and $\delta =$ 3.6 (blue line).

**Figure 3:**
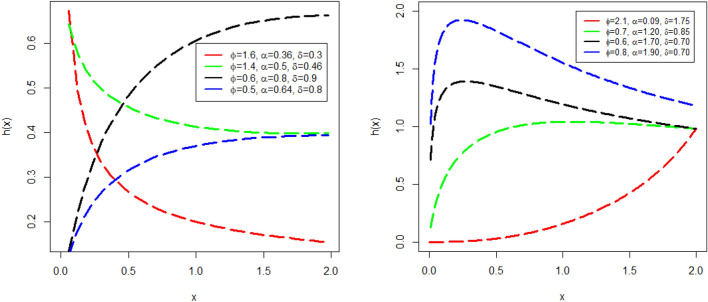
Some different plots of $h(x;\phi ,\mu )$ with different parameters values of NGEP-Wei distribution.

Similarly, some increasing, decreasing, unimodal, and bathtub shape plots of HF $h(x;\phi ,\mu )$ are also visualized in Fig. 3. The plots in left penal of the Fig. 3 of $h(x;\phi ,\mu )$ are obtained with different parameters values (i) $\phi =$ 1.6, $\alpha =$ 0.36, and $\delta =$ 0.3 (red line), (ii) $\phi =$ 1.4, $\alpha =$ 0.5, and $\delta =$ 0.46 (green line), (iii) $\phi =$ 0.6, $\alpha =$ 0.8 and $\delta =$ 0.9 (black line), and (iv) $\phi =$ 0.5, $\alpha =$ 0.64, and $\delta =$ 0.8 (blue line). The plots in right penal of the Fig. 3 of $y(x;\phi ,\mu )$ are obtained with different parameters values (i) $\phi =$ 2.1, $\alpha =$ 0.09, and $\delta =$ 1.75 (red line), (ii) $\phi =$ 0.7, $\alpha =$ 1.20, and $\delta =$ 0.85 (green line), (iii) $\phi =$ 0.6, $\alpha =$ 1.70, and $\delta =$ 0.70 (black line), and (iv) $\phi =$ 0.8, $\alpha =$ 1.90, and $\delta =$ 0.70 (blue line).

## Basic mathematical properties

In the present section, we derive some mathematical properties of the NGEP-X family of distributions. These properties include the QF (quantile function), moments, and MGF (moment-generating function), order statistic, and Residual and Reverse Residual life.

### Quantile function

The QF is also called the inverse of the CDF and is generally used for generating RNs (random numbers) from a distribution. The RNs are usually used for simulation purposes to evaluate the performances of the estimators (or estimation method). Later in section "Simulation", we have implemented this method (i.e., inverse distribution function) for generating RNs from the NGEP-Wei distribution. For the NGEP-Wei distributions, the QF is given by

$$\alpha F\left( {x;\delta } \right)^{2} - \log \left( {1 - u} \right) - 2\log F\left( {x;\delta } \right) - \alpha = 0.$$

where, $0 < u < 1$ and $u$ is the solution of the above expression. The expression can be used to generate random samples or random numbers from any special model of the NGEP-X family of distributions.

### *r*th moment

The moment is an important and useful statistical tool to obtain certain characteristics and features of any model. These characteristics are known as (i) CT (central tendency): which deals with the mean point of a distribution, (ii) MD (Measure of dispersion): which take care of the variance of a model (or measure dispersion among the data), (iii) skewness: which describe the tail behaviour of the model, and (iv) kurtosis: which helps in studying the peakiness of the distribution. For the proposed method, the *r*^*th*^ moment expressed by $\mu_{r}^{\prime }$, is derived as

17 $$\mu_{r}^{\prime } = E\left( {X^{r} } \right) = \int_{ - \infty }^{\infty } {x^{r} \,y\left( {x;\phi ,\mu } \right)dx.}$$

Using Eq. (8) in Eq. (17), we have

18 $$E\left( {X^{r} } \right) = 2\int_{ - \infty }^{\infty } {x^{r} } \,a\left( {x;\mu } \right)A\left( {x;\mu } \right)\left( {e^{\phi } - \phi e^{{\phi A\left( {x;\mu } \right)^{2} }} } \right)dx,$$

Next, using the exponential series in Eq. (18), we get

19 $$\mu_{r}^{\prime } = E\left( {X^{r} } \right) = 2\left( {\sum\limits_{j = 0}^{\infty } {\frac{{\phi^{j} }}{j!}\kappa_{r,1} } - \sum\limits_{m = 0}^{\infty } {\frac{{\phi^{m + 1} }}{m!}\kappa_{r,2m + 1}^{\prime } } } \right),$$

where, $$\kappa_{r,1} = \int_{ - \infty }^{\infty } {\mathop x\limits^{r} } \,a\left( {x;\mu } \right)A\left( {x;\mu } \right)dx$$ and $$\kappa_{r,2m + 1} = \int_{ - \infty }^{\infty } {\mathop x\limits^{r} } \,a\left( {x;\mu } \right)A\left( {x;\mu } \right)^{2m + 1} dx$$.

Furthermore, a simple general expression for the MGF of the NGEP-X random variable X, say $M_{x} \left( t \right)$ , is derived as

$$\begin{aligned} M_{x} \left( t \right) & = \int\limits_{0}^{\infty } {e^{tx} y\left( {x;\phi ,\mu } \right)dx} , \\ M_{x} \left( t \right) & = \sum\limits_{r = 0}^{\infty } {\frac{{t^{r} }}{r!}} \int\limits_{0}^{\infty } {x^{r} y\left( {x;\phi ,\mu } \right)dx} \\ M_{x} \left( t \right) & = \, 2\sum\limits_{r = 0}^{\infty } {\left( {\sum\limits_{j = 0}^{\infty } {\frac{{t^{r} \phi^{j} }}{r!j!}\kappa_{r,1} } - \sum\limits_{m = 0}^{\infty } {\frac{{t^{r} \phi^{m + 1} }}{r!m!}\kappa_{r,2m + 1}^{\prime } } } \right)} . \\ \end{aligned}$$

#### Order statistics

In distribution theory, OS is a very crucial importance. They make their appearance (or role) in the reliability analysis, problems of estimation theory and life testing in a number of ways. They can characterize the lifetime of elements or elements of a reliability system.

Let $X_{1} ,{\text{ X}}_{2} , \, {. }{\text{. }}{\text{. , X}}_{k}$ be a random set of observations of size *k* chosen from NGEP-X family with CDF $Y\left( {x;\phi ,\mu } \right)$ and PDF $y\left( {x;\phi ,\mu } \right)$ given by (7) and (8), respectively. Then the DF (density function) of $y_{r:k} \left( x \right)$ is given by “

20 $$y_{r:k} \left( x \right) = \frac{1}{{B\left( {r,k - r + 1} \right)}}y\left( x \right)\left[ {Y\left( x \right)} \right]^{r - 1} \left[ {1 - Y\left( x \right)} \right]^{k - r} .$$

We express the *1*st order statistic as $X_{1:k} = \min \left( {X_{1} ,{\text{ X}}_{2} , \, {. }{\text{. }}{\text{. , X}}_{k} } \right)$ and the *k*th order statistic as $X_{k:k} = \max \left( {X_{1} ,{\text{ X}}_{2} , \, {. }{\text{. }}{\text{. , X}}_{k} } \right).$ Since, $0 < Y\left( x \right) < 1$ for $x > 0.$ We may utilize the binomial expansion of $\left[ {1 - Y\left( X \right)} \right]^{k - r}$ as follow”

21 $$\left[ {1 - Y\left( x \right)} \right]^{k - r} = \sum\limits_{i = 0}^{k - r} {\left( { - 1} \right)^{i} } \left[ {Y\left( x \right)} \right]^{i} .$$

On using Eq. (21) into Eq. (20), we get

22 $$y_{r:k} \left( x \right) = \frac{y\left( x \right)}{{B\left( {r,k - r + 1} \right)}}\sum\limits_{i = 0}^{k - r} {\left( { - 1} \right)^{i} } \left[ {Y\left( x \right)} \right]^{r + i - 1} .$$

Using Eqs. (7) and (8), in Eq. (22), we obtain the DF of $y_{r:k} \left( x \right)$.

### Residual and reverse residual lifetime

The RL (residual lifetime) and RRL (reverse residual lifetime) offer broader application (or characteristics) in risk management, actuarial measures, biometry, and survival analysis. The RL of the NGEP-X family with a random variable X, say $R_{\left( X \right)} \left( t \right)$ is defined as

$$\begin{aligned} R_{\left( X \right)} \left( t \right) & = \frac{{S\left( {x + t} \right)}}{S\left( t \right)}, \\ R_{\left( X \right)} \left( t \right) & = \frac{{\left( {e^{{\phi A\left( {x + t;\mu } \right)^{2} }} - e^{\phi } F(x + t;\mu )^{2} } \right)}}{{\left( {e^{{\phi A\left( {t;\mu } \right)^{2} }} - e^{\phi } A(t;\mu )^{2} } \right)}},\;\;x \in {\mathbb{R}}. \\ \end{aligned}$$

In addition to the RL, we also obtain the RRL of the NGEP-X family of distributions. The RRL, say $\overline{R}_{\left( X \right)} \left( t \right)$ is given by

$$\begin{aligned} \overline{R}_{\left( X \right)} \left( t \right) & = \frac{{S\left( {x - t} \right)}}{S\left( t \right)}, \\ \overline{R}_{\left( X \right)} \left( t \right) & = \frac{{\left( {e^{{\phi A\left( {x - t;\mu } \right)^{2} }} - e^{\phi } F(x - t;\mu )^{2} } \right)}}{{\left( {e^{{\phi A\left( {t;\mu } \right)^{2} }} - e^{\phi } A(t;\mu )^{2} } \right)}},\;\;x \in {\mathbb{R}}. \\ \end{aligned}$$

## Estimation, experiment and simulation

This section provides a detailed description of the maximum likelihood estimation implemented for obtaining the parameter estimates of the proposed family of distributions. Furthermore, we also conduct a comprehensive Monte Carlo simulation study to assess the performance of the estimators (or the estimation method).

### Maximum likelihood estimation

Several methods of estimation are proposed for obtaining the parameter estimates in the various studies. MLE (Maximum likelihood estimation) is one of the most frequently used methods of estimation. This method furnishes estimators with several important properties and can be used in the construction of confidence intervals as well as other tests for checking statistical significance. For further details about MLEs, please see^19^. This sub-section provides a discussion on the MLEs approach for obtaining the parameter estimates of the NGEP-Wei distributions.

Suppose $x_{1} ,x_{2} ,...,x_{n}$ are the observed values from PDF $y\left( {x;\phi ,\mu } \right)$ given in Eq. (8). Then, the likelihood function corresponding $y\left( {x;\phi ,\mu } \right)$ is expressed by

23 $$L = \prod\limits_{i = 1}^{n} {y\left( {x;\phi ,\mu } \right)} .$$

Now, the log-likelihood function derived by putting Eq. (8) into Eq. (23) and taking the log

24 $$L\left( \Xi \right) = n\log 2 + \sum\limits_{i = 1}^{n} {\log a\left( {x;\mu } \right)} + \sum\limits_{i = 1}^{n} {\log A\left( {x;\mu } \right)} + \sum\limits_{i = 1}^{n} {\log \left( {e^{\phi } - \phi e^{{\phi A\left( {x;\mu } \right)^{2} \,}} } \right)} ,$$

where, $\Xi = \left( {\phi ,\alpha ,\delta } \right)^{T}$. The log-likelihood function can be maximized either directly by using the R package (AdequecyModel), Ox program (subroutine Max BFGS) or SAS (PROC NLMIXED) (see, Doornik^25^ for more reading) or by solving the nonlinear log-likelihood equations obtained by differentiating Eq. (24). So, the partial derivatives of Eq. (24), the, we get

25 $$\frac{\partial L\left( \Xi \right)}{{\partial \phi }} = \sum\limits_{i = 1}^{n} {\frac{{e^{\phi } - \phi A\left( {x;\mu } \right)^{2} e^{{\phi A\left( {x;\mu } \right)^{2} }} - e^{{\phi A\left( {x;\mu } \right)^{2} }} }}{{\left( {e^{\phi } - \phi e^{{\phi A\left( {x;\mu } \right)^{2} }} } \right)}}} ,$$

and

26 $$\begin{aligned} \frac{\partial L\left( \Xi \right)}{{\partial \mu }} & = \sum\limits_{i = 1}^{n} {\frac{{{{\partial a\left( {x_{i} ;\mu } \right)} \mathord{\left/ {\vphantom {{\partial a\left( {x_{i} ;\mu } \right)} {\partial \mu }}} \right. \kern-0pt} {\partial \mu }}}}{{a\left( {x_{i} ;\mu } \right)}}} + \sum\limits_{i = 1}^{n} {\frac{{{{\partial A\left( {x_{i} ;\mu } \right)} \mathord{\left/ {\vphantom {{\partial A\left( {x_{i} ;\mu } \right)} {\partial \mu }}} \right. \kern-0pt} {\partial \mu }}}}{{A\left( {x_{i} ;\mu } \right)}}} \\ & \quad + \sum\limits_{i = 1}^{n} {\frac{{2\phi^{2} A\left( {x_{i} ;\mu } \right)e^{{\phi A\left( {x_{i} ;\mu } \right)^{2} \,}} {{\partial A\left( {x_{i} ;\mu } \right)} \mathord{\left/ {\vphantom {{\partial A\left( {x_{i} ;\mu } \right)} {\partial \mu }}} \right. \kern-0pt} {\partial \mu }}}}{{\left( {e^{\phi } - \phi e^{{\phi \,A\left( {x_{i} ;\mu } \right)^{2} }} } \right)}}} . \\ \end{aligned}$$

Equating the Eq. (25) $\frac{\partial L\left( \Xi \right)}{{\partial \phi }}$ and Eq. (26) $\frac{\partial L\left( \Xi \right)}{{\partial \mu }}$ to zero, and simultaneously solving, yield these expression MLEs of $\phi$ and $\mu$.

### Simulation

To cover the second aim of this section, the performance of the MLEs $\left( {\phi_{MLE} ,\alpha_{MLE} ,\delta_{MLE} } \right)$ of $\left( {\phi ,\alpha ,\delta } \right)$ is assessed by conducting a MCSS (monte Carlo Simulation study). We consider different sample size (i.e., n = 50, 100, 200, 300, 400, 500, 600, 700, 800, 900, and 1000) with different parameters values $\phi =$(0.9, 0.7, 0.8, 0.7), $\alpha =$(1.0, 1.5, 0.7, 1.8), and $\delta =$(1.9, 0.5, 1.2, 0.8). As we have already mentioned the range of $\phi \in {\mathbb{R}}^{ + }$, $\alpha \in {\mathbb{R}}^{ + }$, and $\delta \in {\mathbb{R}}^{ + }$. So, we can choose any values (by default or predefined values of parameters) within their ranges of $\phi$, $\alpha$, and $\delta$ to conduct the simulation study. For each combination of parameters values, the MCSS is repeated 1000 times and the AMLEs (average of MLEs), ABs (average of Biases), and MSEs (mean square error) values are gained. The ABs, and MSEs are calculated using the following expression

$$bias(\Phi ) = \frac{1}{N}\sum\limits_{i = 1}^{N} {\left( {\hat{\Phi }_{i} - \Phi } \right)} ,$$

and

$$MSE(\Phi ) = \frac{1}{N}\sum\limits_{i = 1}^{N} {\left( {\hat{\Phi }_{i} - \Phi } \right)^{2} } ,$$

where, $\Phi = \left( {\phi ,\alpha ,\delta } \right)$. The numerical results for set I = ($\phi = 0.9,\,\alpha = 1.0,\,\delta = 1.9$), and Set II = ($\phi = 0.7,\,\alpha = 1.5,\,\delta = 0.5$) are recorded in Table 1, while the numerical MCSS results for set III = ($\phi = 0.8,\,\alpha = 0.7,\,\delta = 1.2$) and Set IV = ($\phi = 0.7,\,\alpha = 1.8,\,\delta = 0.8$) are presented in Table 2. In Tables 1 and 2, the simulation results are obtained by using the R-script with L-BFGS-B method. Based on the numerical results (or numerical facts) in Tables 1 and 2, we can observe that as the sample size $n$ increase (i.e., $n \to \infty$), the.

- MSE of $\hat{\phi }_{MLE}$, $\hat{\alpha }_{MLE}$, and $\hat{\delta }_{MLE}$ decay to zero.
- MLEs of $\hat{\phi }_{MLE}$, $\hat{\alpha }_{MLE}$, and $\hat{\delta }_{MLE}$ become closer to the true values.
- Biases of $\hat{\phi }_{MLE}$, $\hat{\alpha }_{MLE}$, and $\hat{\delta }_{MLE}$ also decrease.

**Table 1:** MCSS results for NGEP-Wei distribution with different combination of parameters values.

n	Est	Set I:$\phi = 0.9,\,\alpha = 1.0,\,\delta = 1.9$			Set II:$\phi = 0.7,\,\alpha = 1.5,\,\delta = 0.5$		
		AMLEs	MSEs	ABs	AMLEs	MSEs	ABs
50	$\phi$	2.8295152	8.52331816	1.5811746393	0.7399052	2.82293381	0.39448767
	$\alpha$	0.9333573	0.065967868	−0.055722548	1.544058	0.09817217	0.005713591
	$\delta$	1.754639	0.161472844	−0.1740907378	0.4970734	0.00909711	0.006312572
100	$\phi$	2.0309181	5.09024322	1.1309181496	0.5833240	0.51099052	−0.11667602
	$\alpha$	0.9708093	0.043473246	−0.029190672	1.544882	0.04934609	0.044882305
	$\delta$	1.754320	0.134428869	−0.1456804345	0.4992120	0.0020656677	−0.000787979
200	$\phi$	1.6171153	3.33376234	0.7171153082	0.5299882	0.20763766	−0.17001178
	$\alpha$	0.9925023	0.032817710	−0.007497723	1.548448	0.03902823	0.048448144
	$\delta$	1.790207	0.105442784	−0.1097931979	0.4967701	0.0008243972	−0.003229935
300	$\phi$	1.2935387	1.98636160	0.3935386652	0.5911336	0.14895937	−0.10886645
	$\alpha$	1.0170566	0.025652871	0.017056623	1.519749	0.03272702	0.019749008
	$\delta$	1.836130	0.066478316	−0.0638697388	0.4956399	0.0006034432	−0.004360115
400	$\phi$	1.1565709	1.35991763	0.2565708748	0.5738637	0.13477031	−0.12613630
	$\alpha$	1.0163500	0.020806866	0.016350003	1.526217	0.03056997	0.026217321
	$\delta$	1.852868	0.050546110	−0.0471315769	0.4960813	0.0004504960	−0.003918749
500	$\phi$	1.0344630	0.81687494	0.1344630405	0.5701568	0.11278547	−0.12984317
	$\alpha$	1.0221229	0.017025525	0.022122881	1.531690	0.03030522	0.031690076
	$\delta$	1.870636	0.034426115	−0.0293643435	0.4952322	0.0003335911	−0.004767831
600	$\phi$	0.9695984	0.51043941	0.0695984056	0.5983371	0.09322090	−0.10166286
	$\alpha$	1.0217724	0.013865733	0.021772444	1.518970	0.02557863	0.018969779
	$\delta$	1.881736	0.022381627	−0.0182636572	0.4956333	0.0002997004	−0.004366677
700	$\phi$	0.9203701	0.30950644	0.0203701047	0.6053952	0.09029518	−0.09460476
	$\alpha$	1.0256675	0.012718606	0.025667511	1.514795	0.02513799	0.014794894
	$\delta$	1.890928	0.016171932	−0.0090719819	0.4956012	0.0002494730	−0.004398840
800	$\phi$	0.9004115	0.22447142	0.0004114608	0.6085708	0.08545514	−0.09142921
	$\alpha$	1.0269242	0.012243592	0.026924151	1.515382	0.02504993	0.015382435
	$\delta$	1.889684	0.013484865	−0.0103163108	0.4968129	0.0002422502	−0.003187063
900	$\phi$	0.8710536	0.06958742	−0.0289463893	0.6183263	0.07425003	−0.08167368
	$\alpha$	1.0265209	0.009804688	0.026520906	1.514956	0.02192106	0.014956016
	$\delta$	1.896029	0.007033279	−0.0039710725	0.4964915	0.0002091654	−0.003508541
1000	$\phi$	0.8596384	0.03612116	−0.0403616179	0.6229132	0.06381277	−0.07708678
	$\alpha$	1.0273299	0.009903526	0.027329917	1.511150	0.02167760	0.011150494
	$\delta$	1.900566	0.005952839	0.0005656867	0.4957554	0.0001767675	−0.004244554

**Table 2:** MCSS results for NGEP-Wei distribution with different combination of parameters values.

n	Est	Set III:$\phi = 0.8,\,\alpha = 0.7,\,\delta = 1.2$			Set IV:$\phi = 0.7,\,\alpha = 1.8,\,\delta = 0.8$		
		AMLEs	MSE	ABs	AMLEs	MSE	ABs
50	$\phi$	1.3401419	3.73696921	0.540141877	0.8221352	1.60373271	0.348790717
	$\alpha$	0.7209277	0.026167164	0.020927651	1.833487	0.11695117	0.043725520
	$\delta$	1.152840	0.080740908	−0.047160170	0.7954637	0.01193009	0.006672544
100	$\phi$	1.0639405	1.98095765	0.263940510	0.6928009	0.86577761	−0.007199094
	$\alpha$	0.7196219	0.014107102	0.019621861	1.832861	0.08809218	0.032861292
	$\delta$	1.167813	0.026743453	−0.032186898	0.7911665	0.006896347	−0.008833518
200	$\phi$	0.8787327	1.12014754	0.078732678	0.5934661	0.34166207	−0.106533854
	$\alpha$	0.7299946	0.011426791	0.029994553	1.832323	0.06471138	0.032323185
	$\delta$	1.174071	0.016923436	−0.025928824	0.7867408	0.002915682	−0.013259159
300	$\phi$	0.7855351	0.51353181	−0.040098399	0.5541187	0.19567145	−0.145881268
	$\alpha$	0.7252219	0.009306032	0.027827067	1.834145	0.05585333	0.034144703
	$\delta$	1.178937	0.008845356	−0.014837884	0.7932608	0.00169396	−0.006739160
400	$\phi$	0.7137202	0.25206101	−0.086279844	0.5901948	0.14774553	−0.109805244
	$\alpha$	0.7293404	0.008248674	0.029340359	1.821428	0.04697499	0.021427846
	$\delta$	1.190765	0.004827222	−0.009234729	0.7926479	0.001339951	−0.007352076
500	$\phi$	0.7224529	0.19017907	−0.077547090	0.5670105	0.13901290	−0.132989546
	$\alpha$	0.7219769	0.007216719	0.021976925	1.836642	0.04552487	0.036641898
	$\delta$	1.189611	0.003973352	−0.010388667	0.7931329	0.001026925	−0.006867102
600	$\phi$	0.7220863	0.14494077	−0.077913705	0.5780741	0.12905761	−0.121925861
	$\alpha$	0.7223801	0.006567211	0.022380082	1.827314	0.04449885	0.027313535
	$\delta$	1.190422	0.003082486	−0.009577535	0.7908397	0.000945917	−0.009160279
700	$\phi$	0.7127174	0.10754679	−0.087282615	0.5945143	0.09703003	−0.105485702
	$\alpha$	0.7237454	0.006713205	0.023745436	1.821323	0.03990010	0.021323237
	$\delta$	1.192115	0.002415838	−0.007884738	0.7924756	0.000714308	−0.007524425
800	$\phi$	0.7082831	0.09268212	−0.091716874	0.5842262	0.09692024	−0.115773793
	$\alpha$	0.7230103	0.006303908	0.023010301	1.827301	0.03860714	0.027300792
	$\delta$	1.191677	0.002180030	−0.008322607	0.7924884	0.000641495	−0.007511647
900	$\phi$	0.7246462	0.05970638	−0.075353835	0.6033333	0.09024770	−0.096666676
	$\alpha$	0.7184143	0.005301582	0.018414289	1.815633	0.03647963	0.015633414
	$\delta$	1.191563	0.001618962	−0.008437136	0.7930041	0.000597343	−0.006995930
1000	$\phi$	0.7312756	0.05373190	−0.068724370	0.6007523	0.08558905	−0.099247718
	$\alpha$	0.7157047	0.004913919	0.015704749	1.822380	0.03595069	0.022379732
	$\delta$	1.194376	0.001417435	−0.005623963	0.7931987	0.000548591	−0.006801286

## Real life application to coved-19 data sets

Here, we consider two applications from COVID-19 data sets to illustrate the fitting power of the NGEP-Wei distribution. We applied the NGEP-Wei distribution on both data sets and compared its flexibility (or fitting power) with the other rival distributions. The rival distributions of the NGEP-Wei distribution are presented in the following Table 3.

**Table 3:** Rival distributions of the NGEP-Wei distribution.

S. no	Rival or classical distributions	Abbreviations	Author references
1	Alpha Power Transformed Weibull	APTra-Wei	Dey et al.^9^
2	New Reduce Logarithmic Weibull	NRLog-Wei	Liu et al.^26^
3	Kumaraswamy Weibull	Kumar-Wei	Cordeiro et al.^27^
4	Weibull	Wei	Weibull^28^
5	Marshal Olkin Nadarajah Haghigh	MO-NH	Muhammad et al.^29^
6	Gull Alpha Power Weibull	GAP-Wei	Ijaz et al.^30^

The SFs of these rival distributions are the following:

- APTra-Wei distribution
  $$S\left( {x;a,\alpha ,\delta } \right) = 1 - \left( {\frac{{a^{{\left( {1 - e^{{ - ax^{\delta } }} } \right)}} - 1}}{a - 1}} \right)\;\;x \in {\mathbb{R}},$$
- NRLog-Wei distribution
  $$S\left( {x;\phi ,\alpha ,\delta } \right) = \frac{{\log \left( {\phi + 1 - \phi \left( {1 - e^{{ - \alpha x^{\delta } }} } \right)} \right)}}{{\log \left( {1 + \phi } \right)}},\;\;x \in {\mathbb{R}},$$
- Kumar–Wei distribution
  $$S\left( {x;a,b,\alpha ,\delta } \right) = \left( {1 - \left( {1 - e^{{ - \alpha x^{\delta } }} } \right)^{a} } \right)^{b} ,\;\;x \in {\mathbb{R}},$$
- Wei distribution
  $$S\left( {x;,\alpha ,\delta } \right) = e^{{ - \alpha x^{\delta } }} ,\;\;x \in {\mathbb{R}},$$
- MO-NH distribution
  $$S\left( {x;\phi ,\alpha ,\delta } \right) = 1 - \left( {\frac{{1 - e^{{[1 - (1 + \alpha x)^{\delta } ]}} }}{{1 - (1 - \phi )e^{{[1 - (1 + \alpha x)^{\delta } ]}} }}} \right),\;x \in {\mathbb{R}},$$
- GAP-Wei distribution
  $$S\left( {x;\phi ,\alpha ,\delta } \right) = 1 - \left( {\frac{{\phi \left( {1 - e^{{ - \alpha x^{\delta } }} } \right)}}{{\phi^{{\left( {1 - e^{{ - \alpha x^{\delta } }} } \right)}} }}} \right),\;\;x \in {\mathbb{R}},$$

Next, after selecting the rival distributions, we consider certain statistical criteria (i.e., analytical goodness of fit measures) to find out (i.e., fitting power of the competing distributions) the best-suited distribution for the considered COVID-19 data sets. The mathematical formulas (or expression) of this goodness of fits measures (or statistical criteria) are given by

- The CRMS (Cramer–von-Misses)
  $$CRMS = \sum\limits_{i = 1}^{n} {\left[ {A\left( {x_{i} } \right) - \frac{2i - 1}{{2n}}} \right]}^{2} + \frac{1}{12n},$$
- The ANDR (Anderson–Darling)
  $$ANDR = - n - \frac{1}{n}\sum\limits_{i = 1}^{n} {\left( {2i - 1} \right)} \times \left[ {\log A\left( {x_{i} } \right) + \log \left( {1 - A\left( {x_{i - n + 1} } \right)} \right)} \right],$$
- The KS (Kolmogorov–Smirnov)
  $$KS = \sup_{x} \left[ {A_{n} \left( x \right) - \hat{A}\left( x \right)} \right]$$
- The AINC (Akaike information criteria)
  $$AINC = 2p - 2L\left( \Xi \right),$$
- The BINC (Bayesian information criteria)
  $$BINC = p\log (n) - 2L\left( \Xi \right),$$
- The CAINC (Consistent AINC)
  $$CAINC = \frac{2np}{{n - p - 1}} - 2L\left( \Xi \right),$$
- The HQINC (Hannan–Quinn information criteria)
  $$HQINC = 2p\log \left( {\log (n)} \right) - 2L\left( \Xi \right).$$

In the above expressions of the decision criteria, $L\left( \Xi \right)$ is the MLF (maximized likelihood function) evaluated at MLEs, *n *represents the sample size and *p *represents the number of parameters to be estimated in the model. For the NGEP-Wei distribution and Rival distributions, the values of MLEs and the above decision tools (i.e., CRMS, ANDR, KS, AINC, BINC, CAINC, and HQINC) are computed by using R-software with “method = Nelder-Mead” algorithm. In general, among the above-applied distributions to each data set, a distribution with the lowest values of the above goodness of fit measures represents best-suited distribution for the data.

### Analysis of first COVID-19 data sets

The first data set (onward signified by DAST 1) consists of 36 observations and daily new death cases (due to COVID-19) recorded from the period of 10 April to 15 May 2020 in the country of Canada. The data can also be accessible via the link [https://covid19.who.int/]. For mor details about the DS 1, we refer to Almetwally et al.^31^, and Xin et al.^32^. The DAST 1 is: DAST 1 = {3.1091, 3.3825, 2.8636, 3.2218, 4.2781, 4.2202, 2.1901, 2.4141, 1.9048, 2.9078, 3.6426, 3.2110, 3.6346, 2.7957, 1.5157, 2.6029, 3.3592, 2.8349, 3.1348, 2.5261, 1.5806, 2.7704, 3.8594, 4.0480, 4.1685, 3.1444, 3.2135, 2.4946, 3.5146, 4.9274, 3.3769, 6.8686, 3.0914, 4.9378, 3.1091, 3.2823}.

**Figure 4:**
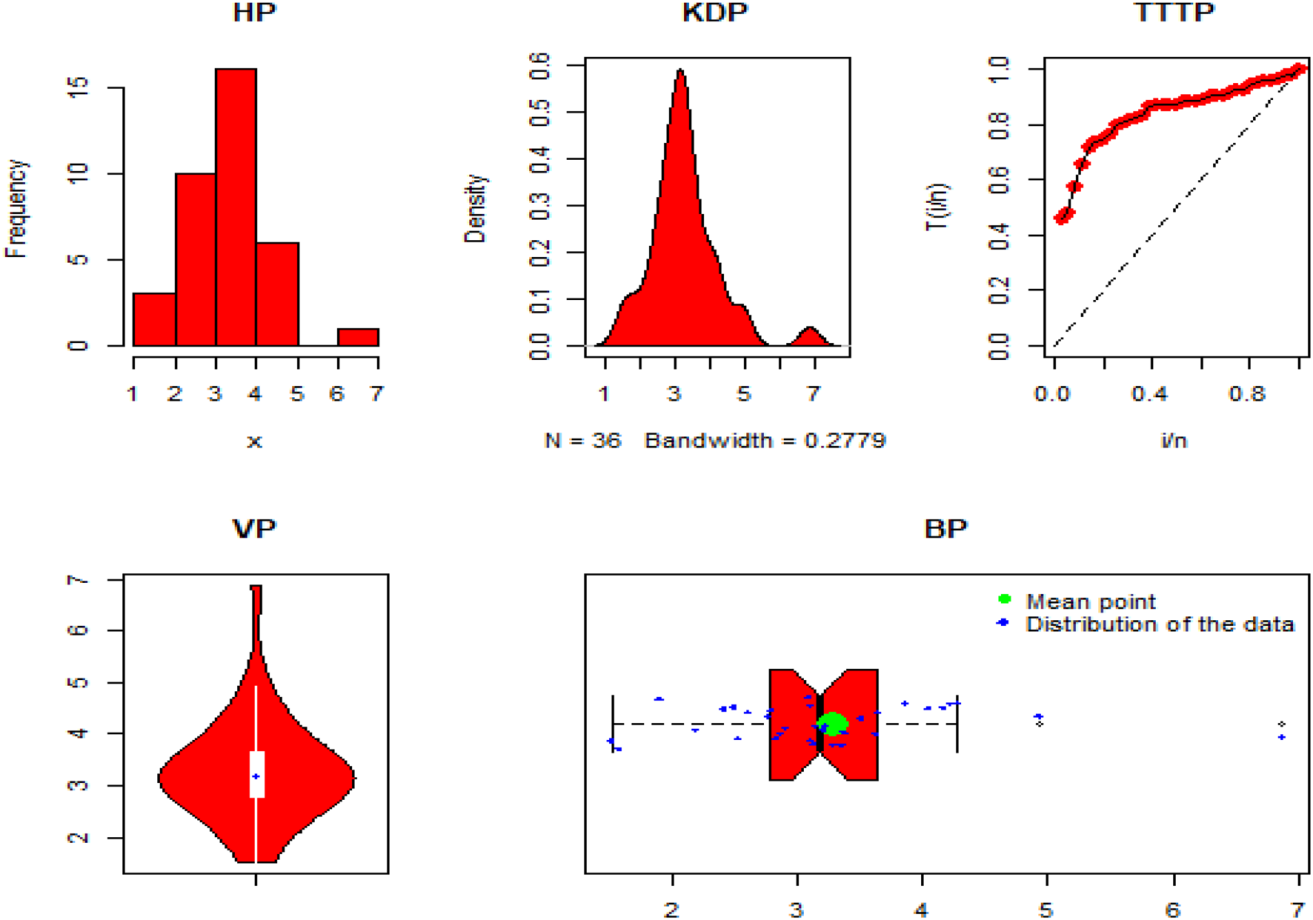
The HP, KDP, TTT-P, VP, and BP of the DATS 1.

Some significant descriptive analysis of DAST 1, are: Min. = 1.516, Max. = 6.869, Mean = 3.282, Q1 (1st quartile) = 2.789, Q2 (2nd quartile or median) = 3.178, Q3 (3rd quartile) = 3.637, Range = 5.3529, variance = 0.9970656, Skewness = 1.213916, and Kurtosis = 6.151625. Additionally, the histogram plot (HP), Kernal density plot (KDP), total-time on test plot (TTT-P), Violin plot, and Box plot (BP) of the DAST 1 are presented in Fig. 4.

**Figure 5:**
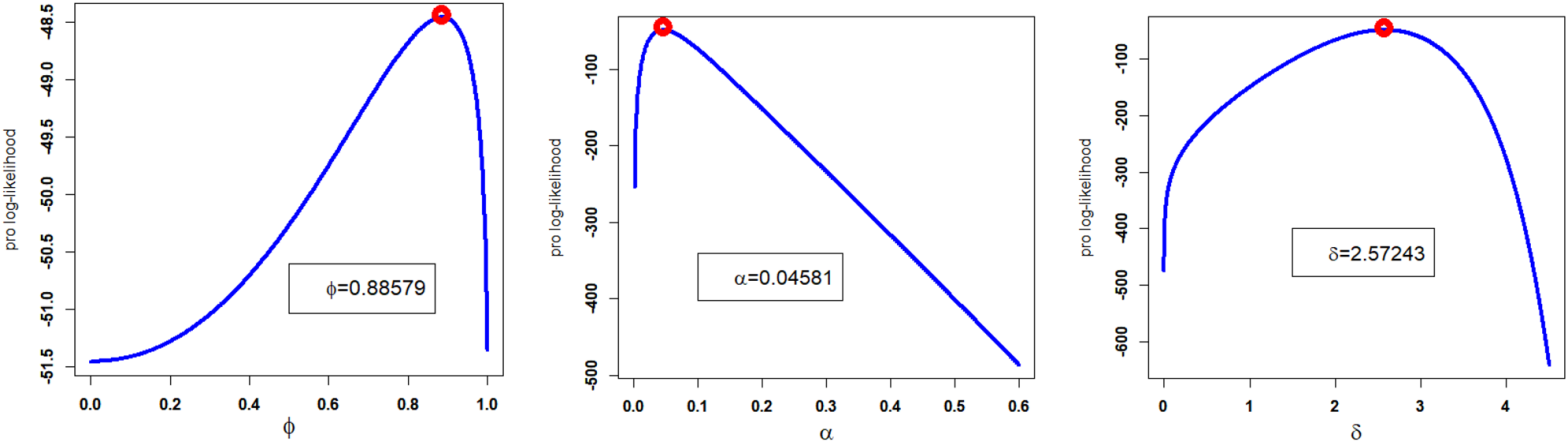
The profile of Likelihood function of $\hat{\phi }$, $\hat{\alpha }$, and $\hat{\delta }$ of NGEP-Wei for DATS 1.

**Figure 6:**
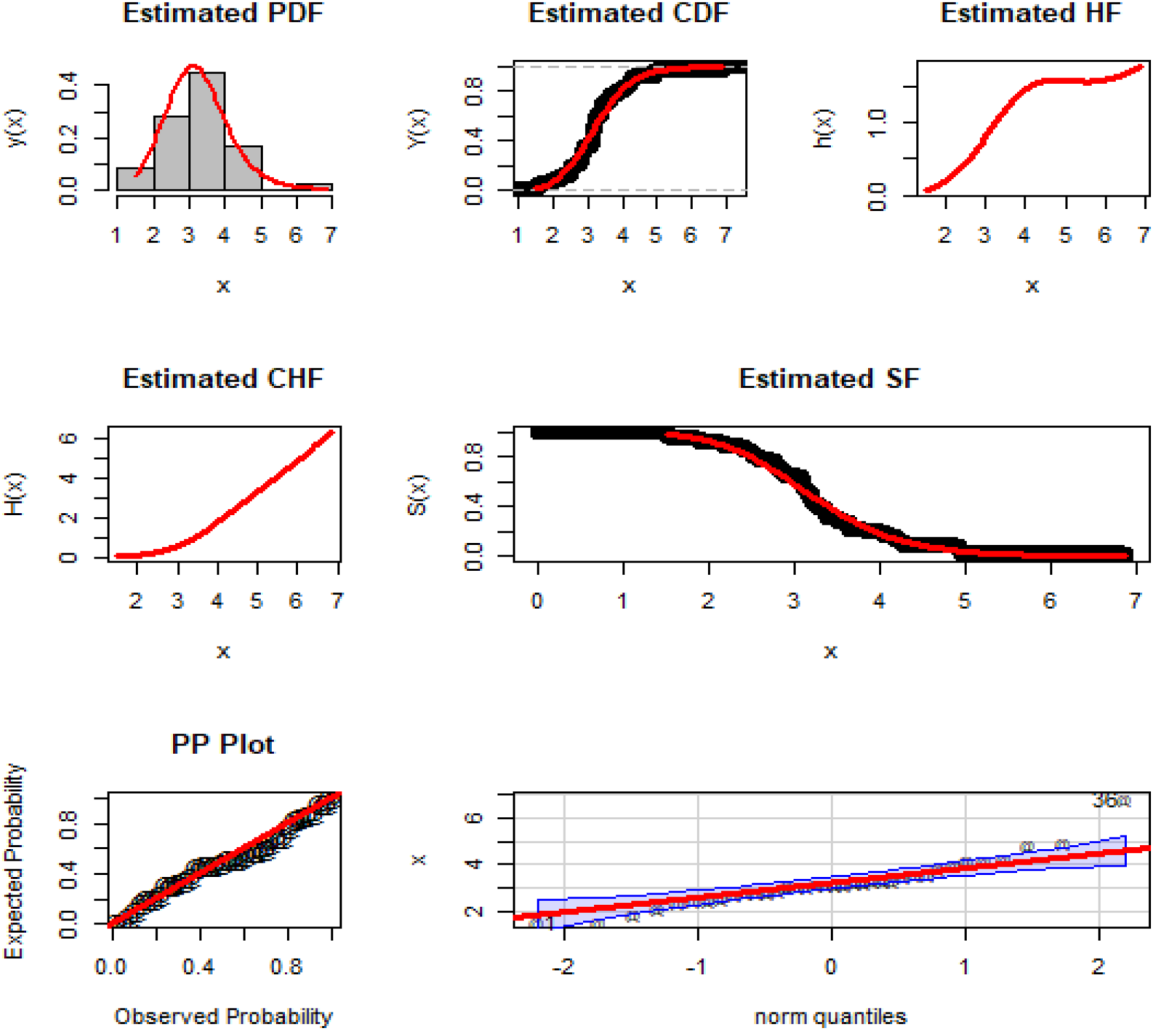
The empirical PDF, CDF, SF, PP-plot and QQ-plot of the NGEP-Wei distribution for DATS 1.

Corresponding to DATS 1, the numerical values of MLEs along with standard errors enclosed in parenthesis of the NFEP-Wei and rival distributions (i.e., $\hat{\phi }_{MLE}$, $\hat{\alpha }_{MLE}$, $\hat{\delta }_{MLE}$, $\hat{a}_{MLE}$, $\hat{b}_{MLE}$) are recorded in Table 4. Furthermore, the numerical values of the goodness of fit measures of the NGEP-Wei and other competitive rival distributions are recorded in Table 5. According to the model selection criteria (goodness of fit measures) in Table 5, the NGEP-Wei distribution provides the best-suited fit with the minimum value of CRMS, ANDR, KS, AINC, BINC, CAINC, and HQINC as compared with rival distributions to the Canada COVID-19 dataset (DATS 1). In other words, based on model selection criteria, we can say that the NGEP-Wei distribution attains reasonable (or satisfactory) fit, which is not sufficiently (or adequately) fitted by the other rival distributions (i.e., APTra-Wei, NRLog-Wei, Kumar-Wei, Wei, MO-NH, and GAP-Wei). Consequently, the NGEP-Wei distribution provides a valuable fit to the DATS 1. Except for the numerical illustration (or comparison) of the NFEP-Wei distribution and other rival distributions, we also presented a visual illustration of the NGEP-Wei distribution. For visual illustration, we plotted the profiles of the log-likelihood function of the $\hat{\phi }_{MLE}$, $\hat{\alpha }_{MLE}$, and $\hat{\delta }_{MLE}$ in Fig. 5. The plots in Fig. 5, we clearly see that the point estimated parameter values of the NGEP-Wei distribution are the maxima. Similarly, the PDF, CDF, SF, PP-plot and QQ-plot for the NGEP-Wei distribution are visualized in Fig. 6. From the graphical illustration in Fig. 6, we can also see that the respective red curve lines of the NGEP-Wei distribution are more close fit to the corresponding empirical objects.

**Table 4:** Estimated MLEs values along with standard errors in parenthesis for DATS 1.

Distributions	$\hat{\phi }_{MLE}$	$\hat{\alpha }_{MLE}$	$\hat{\delta }_{MLE}$	$\hat{a}_{MLE}$	$\hat{b}_{MLE}$
NGEP-Wei	0.88579 (2.04917)	0.04581 (0.00902)	2.57243 (0.36007)	–	–
APTra-Wei	–	2.03279 (0.08744)	0.02448 (0.30557)	3.04691 (126.70663)	–
NRLog-Wei	−0.83524 (0.11319)	0.00259 (0.00068)	4.10087(0.20517)	–	–
Kumar-Wei	–	0.35386 (0.00258)	2.39619 (0.00258)	0.70119 (0.31077)	0.13320 (0.02349)
Wei	–	0.01387 (0.00749)	3.31324 (0.35909)	–	–
MO-NH	22.78807 (1.14637)	11.12585 (4.64358)	0.01742 (0.58032)	–	–
GAP-Wei	0.00265 (0.00336)	0.29942 (0.09719)	1.74019 (0.22120)	–	–

**Table 5:** The analytical measures of the NGEP-Wei and others competitive models for DATS 1.

Distribution	AINC	BINC	CRMS	ANDR	KS	CAINC	HQINC
NGEP-Wei	100.8689	105.6195	0.0747849	0.42072	0.10523	101.6189	102.5270
APTra-Wei	109.0913	113.8419	0.1765308	1.00856	0.14524	109.8413	110.7494
NRLog-Wei	108.3037	113.0543	0.1594273	0.92619	0.16140	109.0537	109.9618
Kumar-Wei	119.4377	125.7717	0.1400811	0.80116	0.24207	120.7280	121.6484
Wei	106.9485	110.1156	0.1729072	0.99175	0.14995	107.3122	108.0539
MO-NH	113.8304	118.5809	0.2434690	1.39362	0.14322	114.5804	115.4884
GAP-Wei	103.4109	108.1615	0.0894868	0.51062	0.13167	103.1609	104.0697

### Analysis of second COVID-19 data sets

The second data set (onward signified by DAST 2) consists of 108 observations. The DAST 2 represents COVID-19 mortality rates and belonging to Mexico for 108 days. This DAST 2 was recently used by Almongy et al.^33^ and suggested a new extended Rayleigh distribution. Observations on the mortality rates were recorded from the period of March 4, 2020 to July 20, 2020. The DAST 2 is: DAST 2 = {8.826, 6.105, 9.391, 14.962, 10.383, 7.267, 13.220, 16.498, 11.665, 6.015, 10.855, 6.122, 6.656, 3.440, 5.854, 10.685, 10.035, 5.242, 4.344, 5.143, 7.630, 14.604, 7.903, 6.370, 3.537, 6.327, 4.730, 3.215, 9.284, 12.878, 8.813, 10.043, 7.260, 5.985 , 6.412, 3.395, 4.424, 9.935, 7.840, 9.550, 3.499, 3.751, 6.968, 3.286, 10.158, 8.108, 6.697, 7.151, 6.560, 2.077, 3.778, 2.988, 3.336, 6.814, 8.325, 7.854, 8.551, 3.228, 7.486, 6.625, 6.140, 4.909, 4.661, 5.392, 12.042, 8.696, 1.815, 3.327, 5.406, 6.182, 1.041, 1.800, 4.949, 4.089, 3.359, 2.070, 3.298, 5.317, 5.442, 4.557, 4.292, 2.500, 6.535, 4.648, 4.697, 5.459, 4.120, 3.922, 3.219, 1.402, 2.438, 3.257, 3.632, 3.233, 3.027, 2.352, 1.205, 3.218, 2.926, 2.601, 2.065, 3.029, 2.058, 2.326, 2.506, 1.923}.

**Figure 7:**
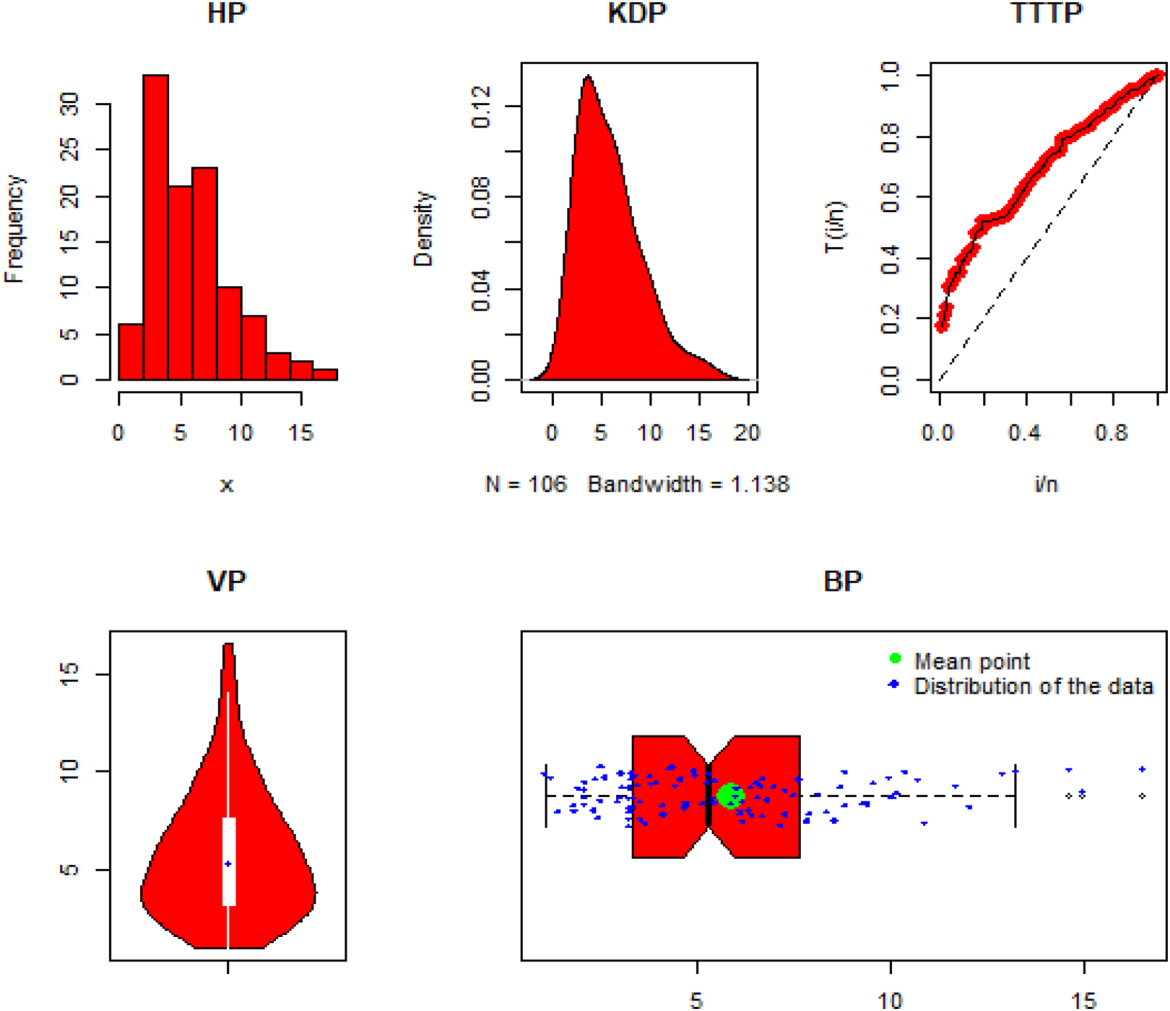
The HP, KDP, TTT-P, VP, and BP of the DATS 2.

Some significant descriptive analysis of DAST 2, are: Min. = 1.041, Max. = 16.498, Mean = 5.822, Q1 (1st quartile) = 3.289, Q2 (2nd quartile or median) = 5.279, Q3 (3rd quartile) = 7.594, Range = 15.457, variance = 10.56173, Skewness = 0.9732453, and Kurtosis = 3.666136. Additionally, the histogram plot (HP), Kernal density plot (KDP), total-time on test plot (TTT-P), Violin plot, and Box plot (BP) of the DAST 2 are presented in Fig. 7.

**Figure 8:**
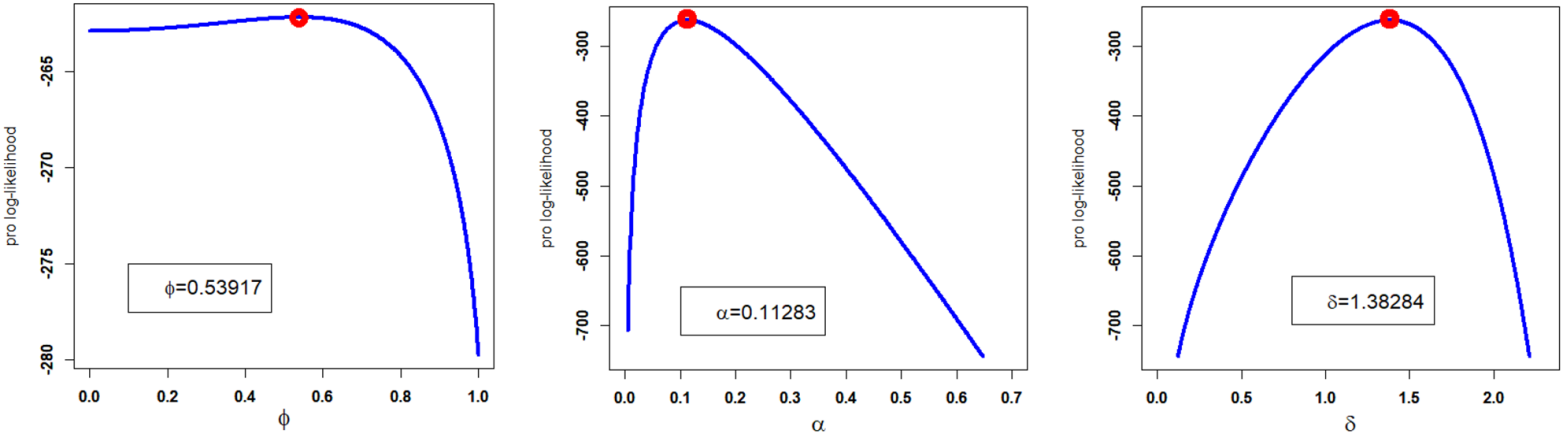
The profile of Likelihood function of $\hat{\phi }$, $\hat{\alpha }$, and $\hat{\delta }$ of NGEP-Wei for DATS 2.

**Figure 9:**
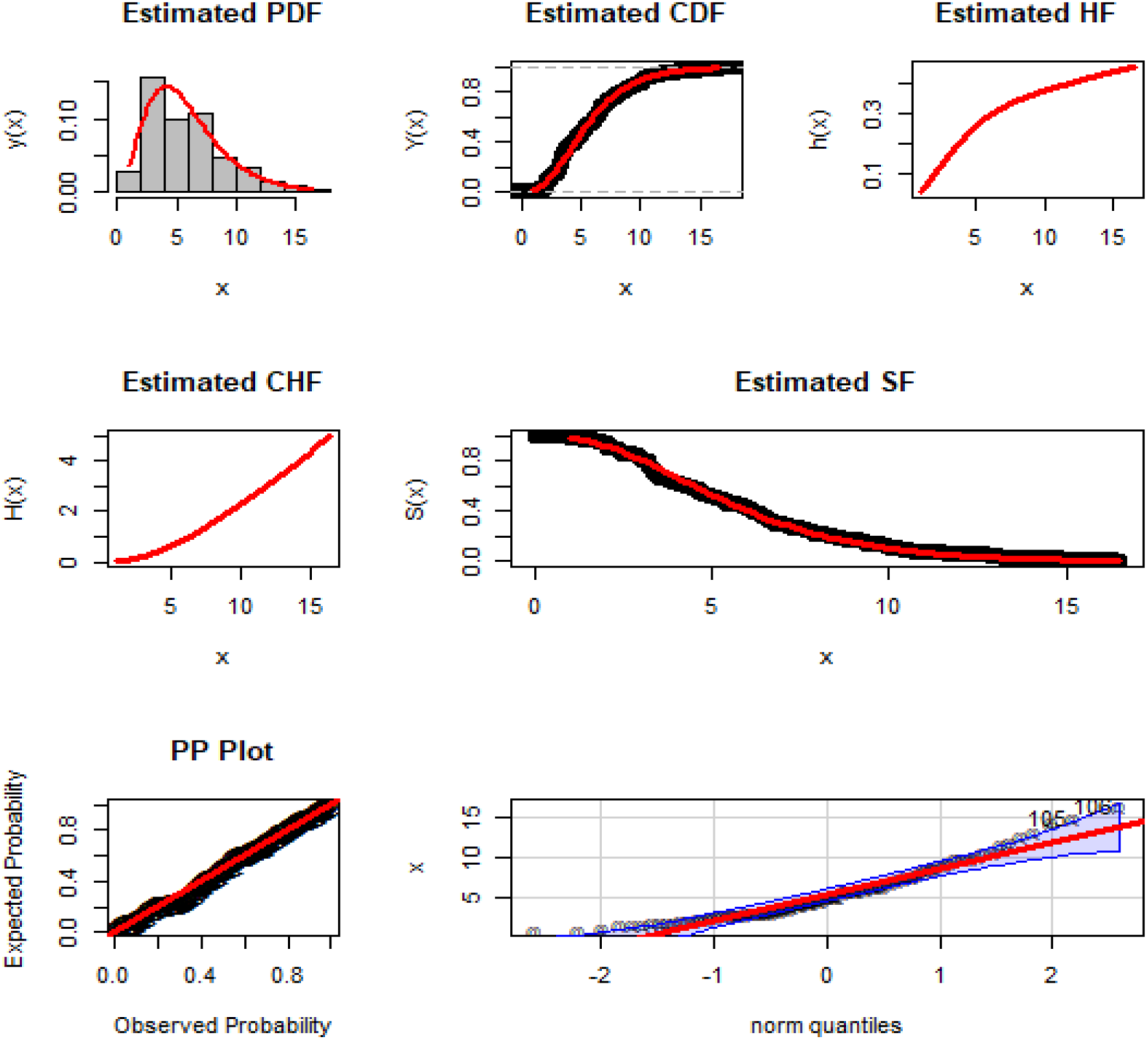
The empirical PDF, CDF, SF, PP-plot and QQ-plot of the NGEP-Wei distribution for DATS 2.

Corresponding to DATS 1, the numerical values of MLEs along with standard errors enclosed in parenthesis of the NGEP-Wei and rival distributions (i.e., $\hat{\phi }_{MLE}$, $\hat{\alpha }_{MLE}$, $\hat{\delta }_{MLE}$, $\hat{a}_{MLE}$, $\hat{b}_{MLE}$) are recorded in Table 6. Furthermore, the numerical values of the goodness of fit measures of the NGEP-Wei and other competitive rival distributions are recorded in Table 7. According to the model selection criteria (goodness of fit measures) in Table 7, the NGEP-Wei distribution provides the best-suited fit with the minimum value of CRMS, ANDR, KS, AINC, BINC, CAINC, and HQINC as compared with rival distributions to the Mexico COVID-19 dataset (DATS 2). In other words, based on model selection criteria, we can say that the NGEP-Wei distribution attains reasonable (or satisfactory) fit, which is not sufficiently (or adequately) fitted by the other rival distributions (i.e., APTra-Wei, NRLog-Wei, Kumar-Wei, Wei, MO-NH, and GAP-Wei). Except for the numerical illustration (or comparison) of the NGEP-Wei distribution and other rival distributions, we also presented a visual illustration of the NGEP-Wei distribution. For visual illustration, we plotted the profiles of the log-likelihood function of $\hat{\phi }_{MLE}$, $\hat{\alpha }_{MLE}$, and $\hat{\delta }_{MLE}$ in Fig. 8. The plots in Fig. 8, we clearly see that the point estimated parameter values of the NGEP-Wei distribution are the maxima. Similarly, the empirical PDF, CDF, SF, PP-plot and QQ-plot are visualized for the NGEP-Wei distribution in Fig. 9. From the graphical illustration in Fig. 9, we can again see that the respective red curve lines of the NGEP-Wei distribution are more close fit to the corresponding empirical objects.

**Table 6:** Estimated MLEs values along with standard errors in parenthesis for DATS 2.

Distributions	$\hat{\phi }_{MLE}$	$\hat{\alpha }_{MLE}$	$\hat{\delta }_{MLE}$	$\hat{a}_{MLE}$	$\hat{b}_{MLE}$
NGEP-Wei	0.53917 (0.50584)	0.11282 (0.03349)	1.38283 (0.10085)	–	–
APTra-Wei		0.10144 (0.10746)	0.00717 (0.00302)	2.24540 (0.14051)	–
FRlog-Wei	1.19154 (0.05608)	0.04610 (0.00166)	1.75252 (0.13879)	–	–
Kumar-Wei	–	0.14472 (0.02699)	1.91841 (0.03279)	1.24081 (0.04679)	0.19223 (0.04993)
Wei	–	0.02675 (0.00829)	1.91968 (0.13893)	–	–
MO-NH	7.26576 (3.32495)	2.20606 (0.55597)	0.02036 (0.01068)	–	–
GAP-Wei	1.91446 (0.53924)	0.01076 (0.00450)	2.14066 (0.14493)	–	–

**Table 7:** The analytical measures of the NEPAW and others competitive models for DATS 2.

Distribution	AIC	BIC	CRMS	ANDR	KS	CIAC	HQIC
NGEP-Wei	530.3143	537.3046	0.0614379	0.360954	0.071092	530.5496	533.5528
APTW	531.5389	539.5292	0.0741368	0.447980	0.076439	531.8403	534.7774
FRlog-Wei	536.4827	544.4730	0.1222831	0.7864775	0.074103	536.7180	539.7212
Kumar-Wei	534.9466	545.6004	0.0869666	0.5548021	0.088029	535.3427	539.2646
Wei	532.5865	537.9134	0.1022121	0.6570378	0.06923	532.7030	534.7455
MO.NH	549.2811	557.2714	0.2304078	1.484616	0.10807	549.5164	552.5196
GAP-Wei	532.2489	540.2392	0.0804400	0.4975332	0.082046	532.4842	535.4874

## Concluding remarks

In this article, we presented a novel generator called a “Novel Generalized Exponent Power-X” family of distributions or in short NGEP-X family. A special sub case of the proposed class by employing the Weibull distribution as a baseline distribution is derived. The special sub-case is named as a Novel Generalized Exponent Power Weibull distribution (NGEP-Wei for short). The density function of the derived model is positively skewed, negatively skewed as well as symmetrical depending on parameter values. Moreover, the hazard function can be monotonically increasing, decreasing, unimodal, and bathtub-shaped. General expressions, for different statistical properties of the proposed class (NGEP-X) have been derived including quantile function, moments, moments generating function, order statistics, residual and reverse residual of lifetime. The maximum Likelihood Estimation method has been used for estimating the model parameters. In addition, a comprehensive MCSS (or simulation) is carried out to assess the performance of the estimators of the proposed model. To prove the efficacy (or fitting power over other classical distributions) of the proposed family of distribution (NGEP-X) based on an NGEP-Wei distribution, we considered two data sets of COVID-19 mortality rates related to the countries of Mexico and Canada. Based on numerical illustration, it is observed that the proposed work outperforms then other widely used existing distributions. For future works, many researchers can use our proposed method to develop new extensions of the existing distributions such as a Novel Generalized Exponent Power Lomax, a Novel Generalized Exponent Power Inverse Lomax, a Novel Generalized Exponent Power Pareto, and a Novel Generalized Exponent Power Lindley that are powerful for representing and predicting real-world phenomena.

## Data Availability

The corresponding author can provide the datasets utilized and/or examined during the present study upon a reasonable request.
